# Evaluation of Women and Underrepresented Racial and Ethnic Group Representation in a General Cardiology Fellowship After a Systematic Recruitment Initiative

**DOI:** 10.1001/jamanetworkopen.2020.30832

**Published:** 2021-01-11

**Authors:** Jennifer A. Rymer, Camille G. Frazier-Mills, Larry R. Jackson, Kevin L. Thomas, Pamela S. Douglas, Andrew Wang, Manesh R. Patel, Anna Lisa Crowley

**Affiliations:** 1 Duke Clinical Research Institute, Durham, North Carolina; 2 Division of Cardiology, Department of Medicine, Duke University Medical Center, Durham, North Carolina

## Abstract

**Importance:**

The proportion of women and underrepresented racial and ethnic groups (UREGs) matriculating into general cardiology fellowships remains low.

**Objective:**

To assess a systematic recruitment initiative aimed at ensuring adequate matriculation of women and UREGs in a general cardiology fellowship.

**Design, Setting, and Participants:**

This quality improvement study took place at a large, tertiary academic medical center and associated Accreditation Council for Graduate Medical Education Cardiovascular Disease fellowship. Participants included cardiology fellowship and divisional leadership and general cardiology fellow applicants to the Duke Cardiovascular Disease Fellowship Program from 2017 to 2019. Data analysis was performed from December 2019 to May 2020.

**Exposure:**

Multipronged initiative that created an environment committed to ensuring equity of opportunity. This included the creation of a fellowship diversity and inclusivity task force that drafted recommendations, which included reorganization of the fellowship recruitment committee, and changes to the applicant screening process, the interview day, applicant ranking process, and postmatch interventions.

**Main Outcomes and Measures:**

The percentage of matriculating and overall women and UREGs before and after the interventions were recorded.

**Results:**

The fellowship received a mean (SD) of 462 (55) applications annually before the interventions (2006-2016) and 611 (27) applications annually after the interventions (2017-2019). Between the 10-year period before the interventions and the 3-year period during the interventions, there was a significant increase in the annual mean (SD) percentage of women (22.4% [2.9%] vs 26.4% [0.07%]; *P* < .001) and UREG applicants (10.5% [1.1%] vs 12.5% [1.9%]; *P* = .01) to the program. Among applicants interviewed, the percentage of women increased from 20.0% to 33.5% (*P* = .01) and that of and UREGs increased from 14.0% to 20.0% (*P* = .01). Before the interventions, a mean (SD) of 23.2% (16.2%) women and 9.7% (7.8%) UREGs matriculated as first-year fellows, whereas after the interventions, a mean (SD) of 54.2% (7.2%) women and 33.3% (19.0%) UREGs matriculated as first-year fellows. The proportion of the entire fellowship who were women increased from a 5-year mean (SD) of 27.0% (8.8%) to 54.2% (7.2%) after 3 years of interventions, and that of UREGs increased from 5.6% (4.6%) to 33.3% (19.0%). Overall, the proportion of applicants in the entire population who were either women or from UREGs increased from 27.8% to 66.7%.

**Conclusions and Relevance:**

After implementing interventions to promote equity of opportunity in the cardiovascular disease fellowship, the percentage of women and UREGs significantly increased in the fellowship over a 3-year time period. These interventions may be applicable to other cardiovascular disease fellowships seeking to diversify training programs.

## Key Points

**Question:**

Is there an association between implementing a systematic recruitment initiative and improved representation of women and underrepresented racial and ethnic groups (UREGs) in a general cardiology fellowship?

**Findings:**

In this quality improvement study, a recruitment initiative was associated with an increase in the percentage of matriculating women, from 23.2% in the 10 years before the initiative to 54.2% in the 3 years after the initiative, and UREG fellows, from 9.7% before the initiative to 33.3% after the initiative. Even greater increases were seen in overall fellowship demographic diversity as the more diverse entering classes diversified the entire fellowship cohort.

**Meaning:**

These findings suggest that a systematic recruitment initiative may be associated with an increase in women and UREG cardiology fellows in a large general cardiology fellowship.

## Teaser

This quality improvement study assess a systematic recruitment initiative aimed at ensuring adequate matriculation of women and underrepresented racial and ethnic groups in a general cardiology fellowship.

## Introduction

The proportions of women and underrepresented racial and ethnic groups (UREGs) (defined as Black, Hispanic, Latinx, or Native American)^1^ in cardiology training programs do not reflect population demographic characteristics. Although the percentage of women internal medicine residents increased from 30.2% to 43.2% between 1991 and 2016,^2^ only 21.3% of cardiovascular fellows in 2016 were women, in stark contrast to 76.9% of geriatrics and 42.9% of hematology-oncology fellows. This may be due to negative perceptions of cardiology among internal medicine residents,^3^ who highlight adverse job conditions, interference with family life, and a lack of existing diverse faculty. The percentage of UREG fellows in cardiology is low (11.6%), which is not substantially different from the percentage of UREGs in internal medicine (13.7%) and other specialties.^4^

In 2017, after the Duke cardiovascular medicine fellowship program matched no women applicants and only 1 UREG fellow, our division prioritized creating a diverse and inclusive class of cardiology trainees guided by Nivet’s principle that diversity drives excellence.^5^ Accordingly, we sought to improve the quality of our training program by creating a more diverse and inclusive fellowship. This quality improvement study describes our objectives, strategies, and interventions.

## Methods

This study meets Duke University Health System institutional review board policies for exemption from review because the study posed no risk to participants; thus, informed consent was not sought. This study complies with each of the 18 points required by the Standards for Quality Improvement Reporting Excellence (SQUIRE) reporting guideline for quality improvement studies.

### Creating an Environment Committed to Change

Our institution’s department of medicine and division of cardiology leadership articulated a clear commitment to increasing inclusivity and diversity in our cardiology fellowship. In response, in 2017, our division created a cardiology fellowship diversity and inclusion task force (TF) to identify and reduce barriers to recruiting diverse classes of trainees.

### Diversity and Inclusion TF

The TF consisted of 17 members (53% women; 24% UREG) of the faculty (10 participants) and fellows (7 participants) who self-identified their interest in TF engagement. A woman associate professor of cardiology (A.L.C.), who was a member of the American College of Cardiology’s Training Council leadership and diversity and inclusion TF, was appointed to lead this TF. This faculty member underwent implicit bias mitigation training.

First, the TF reviewed 10 years of recruitment data to identify unconscious or overt biases. Next, the TF formally surveyed women and UREG applicants, who our program ranked in a position to match but who joined other programs, to obtain their feedback and perceptions of our program. The TF also informally reached out to women and UREG applicants, who our program ranked in a position to match but who joined other programs, via calls or emails to obtain additional feedback about the program. Then the TF created a strength, weakness, opportunity, and threat analysis focusing on increasing diversity in our program. The TF drafted recommendations to division leadership to reduce barriers and increase the recruitment of diverse cardiology fellows. These recommendations were rapidly implemented into the 2017 and future application cycles.

The interventions included in the cardiovascular fellowship diversity recruitment initiative are outlined in the Table. A number of possible interventions were considered, and the final group of interventions for immediate implementation was determined by the TF and division leadership to be those most likely to be effective and also most feasible in our environment. Others, such as hosting second-look interview weekends for women and UREG candidates, were excluded from these initial efforts. TF interventions were recommended for application review, the interview experience, applicant ranking, and post–match day improvements. Specific recommendations for application review were removing any US Medical Licensing Examination score criteria, blinding application reviewers to applicant photos, establishing criteria that women and UREG applicants needed to represent at least 25% of those interviewed to reduce selection bias,^6^ ensuring that each application considered for an interview had 3 independent reviewers, and ensuring that all UREG applicants were independently reviewed by the UREG members of the Fellowship Recruitment Committee (FRC). Importantly, we did not alter (ie, lower) our standard requirements for recruiting applicants. Aside from eliminating US Medical Licensing Examination score criteria, we continued to select applicants to interview who met previously published criteria.^7^ Furthermore, we did not stipulate percentages of ranked or matched applicants.

**Table.** Duke Cardiovascular Fellowship Diversity Quality Improvement Initiative

Overall goals	Strategies	Specific actions
Create an environment committed to change	Establish institutional commitment	Increased commitment by Department of Medicine and Division of Cardiology leadership to increase diversity within the fellowship Formation of diversity and inclusion in recruitment TF to examine barriers to successful recruitment of women and UREG applicants and to plan strategies for changing recruitment strategies
	Diversity and inclusion TF analysis and directives	Performed detailed SWOT analysis and data gathering Solicited feedback from highly ranked applicants who did not match at Duke in prior years Recommended best practices
	Fellowship program leadership changes	Woman PD appointed PD underwent implicit bias mitigation training Journal Club held to educate general cardiology fellows on implicit bias (hosted by A.L.C. and PD) PD served as leadership council member of both the American College of Cardiology Training Council and Diversity and Inclusion TF and leader of division diversity and inclusion TF 4 Associate PDs appointed (50% women; 25% UREGs)
	FRC changes	Restructured to be more inclusive: all faculty welcome but had to attend FRC meetings and interview Ensured at least 33% of the FRC were women faculty and at least 20% were UREGs
Deployment of interventions	Recruitment: application review changes	Removed US Medical Licensing Examination score criteria Blinded applicant reviewers to applicant picture Established criteria for diverse applicant pool with goal for increased proportion of applicants interviewed to be women and UREGs (aimed for at least 25% each) Ensured each applicant considered for an interview had 3 independent application reviewers In addition, all UREG applications were independently reviewed by all UREG members of the Fellowship Recruitment Committee
	Recruitment: interview day changes	Scheduled current women and UREG fellows to attend preinterview dinners to meet with women and UREG applicants Solicited information from applicants about area of clinical and research interests and list of faculty they would like to meet Highlighted the diversity of institutional leadership and success of women and UREG cardiology faculty Paired women applicants with at least 1 women interviewer and another interviewer in area of interest or potential mentor Paired UREG applicants with at least 1 UREG interviewer and another interviewer in area of interest or potential mentor PD interviewed all applicants Collaborated with Department of Medicine Minority Recruitment and Retention Committee to identify trainee development opportunities and resources at Duke in education, research, and mentorship and handed out brochure during interview Scheduled end of interview day informal receptions with fellowship leadership, women fellows, and women and UREG applicants to discuss any remaining questions
	Recruitment: applicant ranking changes	Discussed top candidates with both general FRC and specifically women and UREG faculty and fellows to ensure inclusivity and diversity in our highly ranked applicants
Evaluation and continued improvement	Postmatch interventions	Promoted our successes throughout the institution with regard to increased recruitment of women and UREG applicants Ensured strong mentorship for women and UREG fellows who matched at our institution Held division journal clubs to disseminate information on the benefits of diversity in medicine Continued to obtain feedback from applicants who were ranked to match but did not as to reasons they did not select our program Fostered relationships with Duke School of Medicine students and Internal Medicine residents to mentor and develop a pipeline of excellent & diverse applicants Collaborated with SOM initiatives to improve diversity in faculty recruitment

Abbreviations: FRC, Fellowship Recruitment Committee; PD, program director; SOM, School of Medicine; SWOT, Strength, Weakness, Opportunity, and Threat; TF, task force; UREG, underrepresented racial and ethnic groups.

Specific interview experience interventions included having women and UREG fellows attend the preinterview dinner and lunches to meet applicants and answer questions, highlighting the institution’s commitment to support a diverse fellowship, faculty, and leadership team, soliciting information from applicants a priori regarding their area of clinical or research interest and faculty they would like to meet, scheduling UREG and women applicants to meet with UREG and women faculty interviewers in their areas of interest, and hosting informal receptions at the end of the interview day for women and UREG applicants with division faculty. An additional review of UREG and women applicants was performed to ensure that no qualified applicant was excluded. After the match, our program continued our efforts to support the matched women and UREG fellows with dedicated mentorship and collaborating with our Department of Medicine’s Minority Recruitment and Retention Committee to offer educational and research resources to enable success. Finally, we fostered relationships with Duke School of Medicine (SOM) students and Internal Medicine (IM) residents to mentor and develop a pipeline of excellent and diverse applicants.

The TF also suggested efforts to increase the inclusivity of our fellowship to all applicants. These efforts included ensuring availability and accessibility of lactation rooms for our trainees, promoting the availability of 3 childcare centers on the Duke campus in our recruitment materials, and partnering with our institution’s Graduate Medical Education and Health system to successfully implement 6 weeks of paid parental leave for all trainees.

### Subsequent Modifications

After the initial intervention year, several additional changes occurred that were not part of the original TF recommendations. First, there were CV fellowship program leadership changes (2018). A new program director (PD) was selected who was the former head of the fellowship diversity and inclusion TF and was the first woman PD for this fellowship. In addition, the PD and leadership supported 4 assistant PDs who were 50% women and 25% UREGs and represented several cardiovascular subspecialties. Second, the first woman cardiology chief fellow was peer elected for 2018 to 2019 (J.A.R.). She was a TF member who helped implement changes during her tenure. Third, there were FRC changes. After reviewing the TF findings, the FRC was restructured to be more inclusive, with all members of the cardiovascular faculty and fellows invited to participate. From 2016 to 2019, FRC membership increased from 28 to 51. The number of women nearly doubled (from 9 to 17) from 2016 to 2019 but the percentage (33%) was unchanged because of the increased size of the committee. The number of UREG members increased from 2 (7%) to 11 (22%).

### Feedback on Initiatives

After our interventions, we assessed for differences in medical knowledge and time to achieve milestones between men and women and UREG and non-UREG fellows, comparing within-fellowship class data. We used in-training examination scores, time to achieve milestone competencies, and review of Accreditation Council for Graduate Medical Education (ACGME) annual faculty and fellow program surveys.

### Statistical Analysis

Results are presented as percentage or mean (SD) where indicated. Comparisons between the data in the 5 to 10 years before the interventions and the 3-year data after the interventions are made using an unpaired, 1-sided *t *test with an a priori *P* < .05 performed using Excel for MAC version 16.4 (Microsoft). Data analysis was performed from December 2019 to May 2020.

## Results

During the 10-year period before the interventions (2006-2016), we received a mean (SD) of 462 (55) applications annually, which was not significantly different from the mean (SD) number of applications (440.6 [15.4] applications) among all US ACGME cardiovascular disease fellowship programs.^8^ During the 3-year intervention application cycles (2017-2019), we received 25% more applications annually (mean [SD], 611 [27] applications; *P* < .001). This increase was also significantly more than the increase in the number of applications (mean [SD], 505.3 [15.9] applications) among all US ACGME cardiovascular disease fellowship programs through 2020 (*P* = .002).^8^ Similarly, there was a significant increase in the annual percentage of women (mean [SD], 22.4% [2.9%] vs 26.4% [0.07%]; *P* < .001) and UREG applicants (mean [SD], 10.5% [1.1%] vs 12.5% [1.9%]; *P* = .01) to our program between the 10-year period before the interventions and the 3-year period during the interventions.

Before our interventions, the annual percentage of women applicants to our program was not significantly different from the mean number of women applicants (mean [SD], 22.4% [2.9%] vs 21.7% [2.3%]) among all US ACGME cardiovascular disease fellowship programs.^8^ However, after our interventions, we received a significantly higher percentage of applications from women compared with the percentage from all US ACGME fellowship programs (mean [SD], 26.4% [0.07%] vs 23.1% [0.01%]; *P* = .004).^8^ Among UREGs, we did not receive significantly different percentages of applications compared with the percentages received from all US ACGME fellowship programs before (mean [SD], 10.5% [1.1%] vs 9.5% [1.0%]) and after (mean [SD], 12.5% [1.9%] vs 10.2% [1.6%]) our interventions.^8^

We interviewed the same percentage (10%) of all applicants because our number of matched positions (8 positions) did not change during each year of the interventions. Notably, of those interviewed, the percentage of women increased from 20.0% to 33.5% (*P* = .01) and that of UREGs increased from 14.0% to 20.0% (*P* = .01) after the interventions. We also observed that the number of UREG and women applicants who were ranked in positions 1 to 20 on our National Resident Matching Program submitted rank list increased from means (SDs) of 0.7 (0.8) and 2.0 (1.0), respectively, before the interventions to 3.7 (3.0) and 6.7 (2.3), respectively, after the interventions.

During the 10 years preceding the interventions, a mean (SD) of 23.2% (16.2%) women and 9.7% (7.8%) UREGs matriculated as first-year fellows. In the first application cycle following the interventions, 4 women fellows (50%) and 1 UREG fellow were recruited in a class of 8 general cardiology fellows. During the second application cycle, 5 women (62.5%) and 4 UREGs (50.0%) were recruited. The third and most recent application cycle recruited 4 women (50.0%) and 3 UREG fellows (37.5%). Ultimately, we matriculated a mean (SD) of 4.3 (0.58) women (vs 1.9 [1.4] women) and 2.7 (1.5) UREG (vs 0.8 [0.6]) applicants per year during the 3-year period of the interventions, or a mean (SD) of 54.2% (7.2%) women vs the prior 10-year mean (SD) of 27.0% (8.8%) (*P* = .005) and 33.3% (19.0%) UREG vs the prior 10-year mean (SD) of 5.6% (4.6%) (*P* = .003). Overall, the proportion of applicants in the entire population who were either women or from UREGs increased from 27.8% to 66.7%. As of July 2020, the 3-year Duke general cardiovascular medicine fellowship program (24 participants) was 54.2% women, 33.3% UREG, and 66.7% either. The results of the interventions are demonstrated in the Figure. Results of the 2021 class match, which became available as this article was going to press, show continued high diversity of incoming Duke fellows, with 50% women, 40% UREGs, and 60% women or UREGs.

**Figure.**
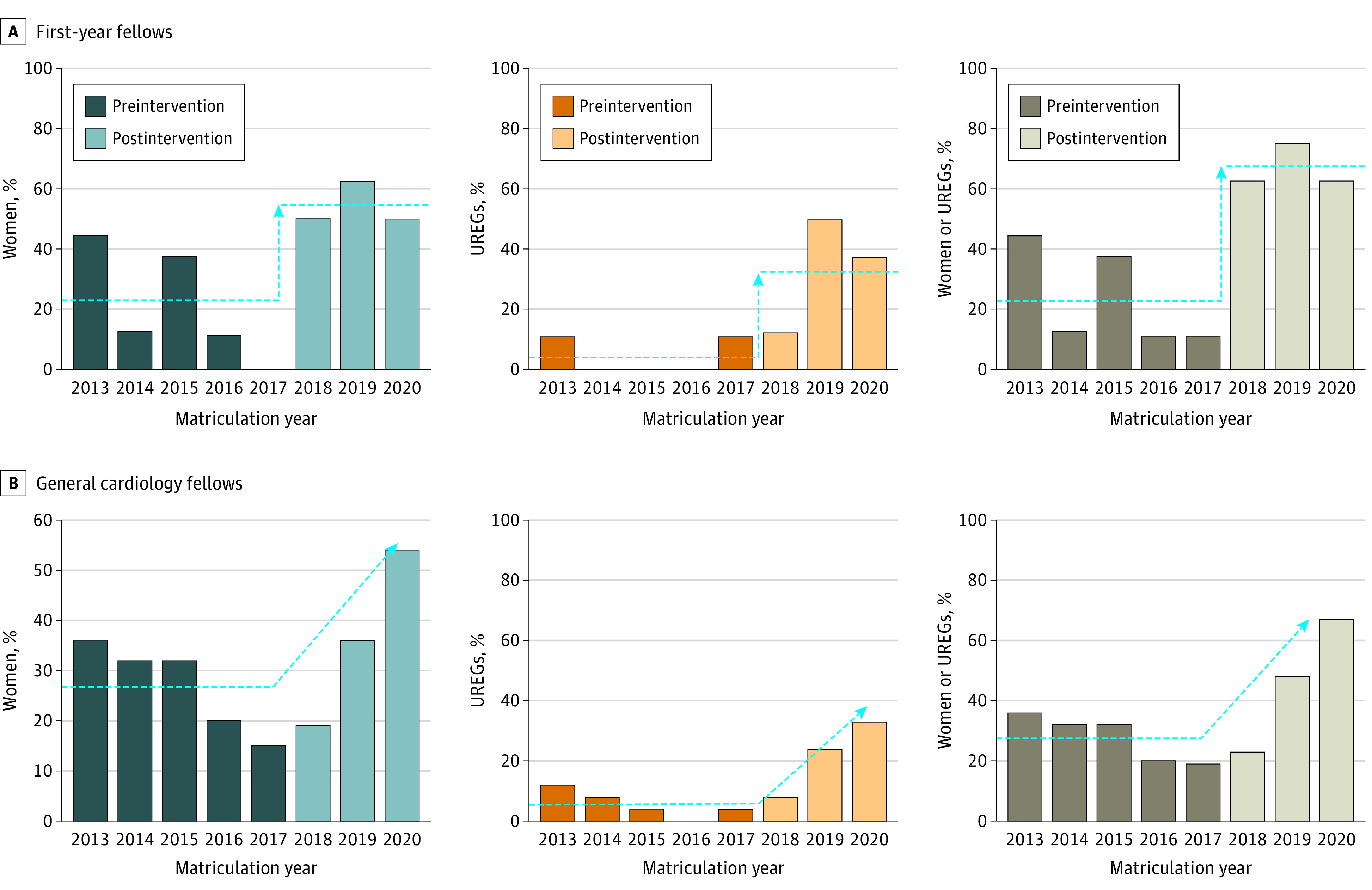
Cardiology Fellows Who Self-identified as Women or Members of an Underrepresented Racial and Ethnic Group (UREG) Who Matriculated Into a Cardiology Program Before and After the Intervention Panel A shows data for first-year fellows, and panel B shows data for all general cardiology fellows. If a fellow self-identified as a woman and a UREG, that fellow was only counted once in the total category. In panel A, the dashed lines over the preintervention and intervention years illustrate the mean percentage of fellows who matriculated during that 5- or 3-year period. The arrow indicates the time period during which the intervention began. There were 9 first-year fellows in 2013, 2016, and 2017 and 8 first-year fellows in 2014, 2015, 2018, 2019, and 2020. In panel B, the dashed line over the preintervention years illustrates the mean percentage of fellows over the 5-year period and the arrows show the cumulative change of successive matriculating classes of more diverse fellows. There were 25 fellows in 2013, 2014, 2015, 2016, and 2019, 26 fellows in 2017 and 2018, and 24 fellows in 2020.

Additionally, of 17 matriculating Duke UREG general cardiology fellows from 2007 to 2020, 1 (5.8%) was a Duke SOM graduate, 8 (47.0%) were Duke IM residency graduates, and 8 (47.0%) were either a Duke SOM or IM residency graduate. Of 33 matriculating Duke women general cardiology fellows from 2007 to 2020, 4 (12.1%) were Duke SOM graduates, 9 (27.2%) were Duke IM residency graduates, and 10 (30.3%) were either a Duke SOM or IM residency graduate.

During the last 2 years, increasing numbers of women general cardiology fellows have gone on to pursue subspecialty advanced cardiology fellowships, including interventional cardiology, advanced heart failure and transplantation, and adult congenital heart disease. Notably, in the past year, 2 women general fellows have pursued training in our interventional cardiology fellowship (of a total of 3 interventional cardiology fellows).

Furthermore, to assess for differences in the medical knowledge and speed of progress through training among fellows, we used 2 sources: in-training examination scores and time to achieve competency milestones. Fellows’ scores for first-time examination takers after the interventions during 2018 and 2019 (19 fellows for both years) did not demonstrate a significant difference between the scores for 5 women and 15 men (mean [SD] score, 72.8 [8.0] points vs 67.4 [8.8] points). Because only 2 UREG fellows took this examination, we cannot make statistical comparisons. However, neither score was a statistical outlier. There was no significant difference between men and women and UREG and non-UREG fellows in aggregate in the time to achieve milestone competency levels.

In addition, we examined our results on the 2019 to 2020 ACGME survey diversity and inclusion questions to assess objective feedback on our program. The 2019 to 2020 ACGME survey included diversity and inclusion questions. Our program means exceeded the specialty means for all the questions in this domain on the fellow survey, including preparation for interaction with diverse individuals, program fosters inclusive work environment, and diverse fellow recruitment and retention. Our program means matched the specialty means for the questions on efforts to recruit diverse fellows and efforts to retain diverse fellows on the faculty survey. In addition, 95% of our faculty respondents indicated that they participated in efforts to recruit diverse fellows, and 100% indicated that they participated in activities to enhance professional skills in “contributing to an inclusive clinical environment.”

We note some additional benefits to our initiative. Our faculty and fellows were eager to help and willingly contributed their time and expertise. We were able to implement all of our initiatives without significant additional expense compared with our recruitment process before our interventions. Furthermore, other Duke fellowship programs looked to our example and asked to implement our strategy to their programs. We are unaware of any specific problems, failures, or negative unintended consequences.

## Discussion

To promote equity of opportunity in our ACGME cardiovascular disease fellowship training program, we instituted a multiyear initiative that was associated with more than doubling the numbers of women and UREG cardiology fellows compared with the prior 10 years. We attribute this success to multipronged, intensive interventions including institutional commitment, thoughtful planning, reorganization of our program leadership and recruitment committee, changes to our recruitment interview processes, and, during recruitment and selection, highlighting our commitment to diversity and reducing implicit bias. We are able to report a significant increase in both women and UREG representation in a cardiology fellowship training program over a short period of 3 years, which may have resulted, in part, from a comprehensive fellowship diversity quality improvement initiative.

Cardiology is not a diverse profession, with underrepresentation of women and Black, Latinx, and other groups.^2^ The American College of Cardiology, American Heart Association, and other societies have recognized this as a problem for the profession that limits access to talent and impacts the care of an increasingly diverse patient population.^9^ The goal of a more diverse profession can be accomplished only by attracting a broader range of trainees. Structural and unconscious biases are prevalent and difficult to counteract, as described in individual case studies of cardiology fellowship applicants by Duvernoy et al.^10^ In business and academia, there is nearly universal recognition that focused efforts are needed to increase diversity; this is likely also true in cardiology training programs.^6,9,10,11^

Our results differ from others^11^ in that we were able to rapidly incorporate a comprehensive set of quality improvement changes and demonstrate that these changes were associated with a significant and sustained increase over a 3-year-period in the women and UREG fellows. In support of this, there are no differences in the in-training examination scores between women and men fellows and between UREG and non-UREG fellows of trainees recruited during the intervention period.

Other institutions have recognized the imperative to promote equity of opportunity and have instituted efforts to successfully increase UREG recruitment. For example, like Auseon and colleagues,^11^ we made promoting equity of opportunity a priority. As recommended by Duvernoy et al,^10^ we emphasized our program’s commitment to mentorship and primed the pipeline with outstanding women and UREG applicants from our own medical school and residency. Furthermore, in mitigating implicit bias, we recognized the need for training and applicant and interview pools of at least 25% of the target group.^6^ Thus, the percentage of women we interviewed increased from 20.0% to 33.5% and that of UREGs increased from 14.0% to 20.0%. To further decrease bias, our 3 initial application reviewers were blinded to applicant photos.

Our results that approximately 50% of UREG cardiology fellows and approximately 30% of women fellows were graduates of either Duke SOM or IM residency highlight the importance of a strong pipeline. Our ability to recruit and retain top Duke applicants was possible, in part, because of initiatives during Duke medical school and residency. In particular, Duke’s Minority Recruitment and Retention Committee (C.G.F.M. and K.L.T.) provides career mentoring, leadership development, and social networking for minority faculty and trainees. Specific trainee-directed educational and research initiatives that our current fellows from Duke benefited from include the visiting student-clinical scholar program, Clinical Translational Science Award TL1 research supplement for medical students, Medicine Endeavor to Nurture Trainees to Research Success supplemental mentorship program, and Duke Center for Research to Advance Healthcare Equity grants. The latter is one of 12 centers of excellence funded by the National Institute of Minority Health and Health Disparities to improve minority health and reduce health disparities. Furthermore, the Duke SOM recently added a Cultural Determinants of Health Disparities curriculum, which will provide an excellent foundation for our future fellows in the coming years. Finally, the cardiology consultation and the cardiac intensive care rotations, which include medical students and residents, have added more dedicated teaching opportunities, such as a faculty-trainee daily morning report and a weekly ICU faculty didactic session, respectively.

Although it was not a guide for our initiative, because it was published this year, we note that our efforts are highly aligned with the 5-point inclusive recruitment framework suggested by Gonzaga and colleagues.^12^ Our initiative included actionable steps in each of these 5 domains: setting diversity as a priority, seeking out candidates, implementing inclusive recruitment practices, investing in trainee success, and building the pipeline. Our success provides considerable evidence supporting their constructs.

There are a number of other potentially relevant factors that were not addressed by our intervention, such as the important role of also recruiting, retaining, and advancing a diverse faculty. These individuals are needed to demonstrate a divisional commitment to diversity, to serve as role models, and to mentor an increasing number of diverse trainees. Furthermore, national efforts highlighting the need for increased diversity in cardiology may have helped gain support and traction for our interventions.

### Future Directions

Future educational interventions and areas for study include expanding implicit bias training to more faculty, expanding core curricula to include lectures on racial and ethnic disparities, and comprehensively assessing the association between the initiation of individual interventions on recruiting more diverse fellowship classes. In addition, assessing the association between deepening pipeline and faculty directed initiatives such as ADVANCE-UP (Academic DeVelopment, Advocacy, Networking, Coaching and Education for Underrepresented Populations; developed by coauthor K.L.T.) on improved recruitment and retainment of diverse faculty is an area of active focus at Duke.

### Limitations

Although our findings are significant, we have a few limitations. First, we cannot determine whether our interventions were associated with the overall 25% increase in applications to our program. However, we observed that the mean increase in the percentage of UREG and women applicants to all ACGME internal medicine residencies (14.0% to 15.2% and 43.3% to 43.7%, respectively) and cardiovascular disease fellowships (9.5% to 10.2% and 21.7% to 23.1%, respectively) did not increase significantly from 2015 to 2019.^13^ Second, our interventions were comprehensive and specific to our institution. Components may not be feasible in all training programs or environments. Third, given the nature of the present study, we were unable to identify the individual benefit of any of the specific components of the initiative.

## Conclusions

This study found that a multipronged, comprehensive quality improvement initiative with multiple strategic and concurrent interventions was associated with significant, immediate improvements in the diversity of our cardiology fellowship program. These results were sustained over a 3-year period with an overall fellowship cohort that is majority women and UREGs. Our experience can serve as a model to other programs seeking to increase diversity of their trainees.
